# Drug delivery for neuronopathic lysosomal storage diseases: evolving roles of the blood brain barrier and cerebrospinal fluid

**DOI:** 10.1007/s11011-021-00893-3

**Published:** 2022-01-28

**Authors:** Yuji Sato, Kohtaro Minami, Toru Hirato, Kazunori Tanizawa, Hiroyuki Sonoda, Mathias Schmidt

**Affiliations:** Research and Development, JCR Pharmaceuticals, Ashiya, Hyogo Japan

**Keywords:** Lysosomal storage disease, Neuronopathic mucopolysaccharidosis, Blood-brain barrier, Cerebrospinal fluid, Neurodegeneration, Enzyme replacement therapy, Transcytosis

## Abstract

Whereas significant strides have been made in the treatment of lysosomal storage diseases (LSDs), the neuronopathy associated with these diseases remains impervious mainly because of the blood-brain barrier (BBB), which prevents delivery of large molecules to the brain. However, 100 years of research on the BBB since its conceptualization have clarified many of its functional and structural characteristics, spurring recent endeavors to deliver therapeutics across it to treat central nervous system (CNS) disorders, including neuronopathic LSDs. Along with the BBB, the cerebrospinal fluid (CSF) also functions to protect the microenvironment of the CNS, and it is therefore deeply involved in CNS disorders at large. Recent research aimed at developing therapeutics for neuronopathic LSDs has uncovered a number of critical roles played by the CSF that require further clarification. This review summarizes the most up-to-date understanding of the BBB and the CSF acquired during the development of therapeutics for neuronopathic LSDs, and highlights some of the associated challenges that require further research.

## Introduction

Lysosomal storage diseases (LSDs) are progressive inherited disorders of the metabolism, in which, due to a genetic deficiency of lysosomal enzymes, substrates remain unmetabolized and accumulate inside the organelles of the endosomal-autophagic-lysosomal system. About two-thirds of patients with LSDs exhibit complex central nervous system (CNS) dysfunctions. Such neuronopathic LSDs include mucopolysaccharidoses (MPS; in particular MPS I, II, III, and VII, also known as neuronopathic MPS), sphingolipidoses, mucolipidoses, oligosaccharidoses, multiple sulfatase deficiency, and neuronal ceroid lipofuscinoses (Sun 2018; Pará et al. 2020).

The recent advent of novel treatments for LSDs (e.g. enzyme replacement therapy [ERT], hematopoietic stem cell transplantation, and gene therapy) has benefited patients by alleviating their symptoms and improving prognoses (Scarpa et al. 2017; Beck 2018; Poswar et al. 2019). However, the multifaceted neurological and psychiatric impairments observed in patients with LSDs (e.g. developmental delay, seizures, cognitive dysfunction, memory deficits, behavioral abnormalities, hydrocephalus, acroparesthesia, motor weakness, gait disturbance, and extrapyramidal signs) have unfortunately defied these therapeutic advances, compromising patients’ quality of life, and limiting long-term survival (Edelmann and Maegawa 2020). This is primarily because the blood-brain barrier (BBB) prevents large therapeutic molecules from entering the brain and, therefore, from exerting any effect on it. For this reason, while ERT significantly alleviates the somatic symptoms of patients with neuronopathic MPS, for example, it is ineffective against their CNS disorders. Drug delivery to the CNS is, therefore, a critical hurdle to overcome in addressing untreatable severe CNS disorders in patients with LSDs (Giugliani et al. 2018a).

The BBB and the cerebrospinal fluid (CSF) both play critical roles in preserving the intricate structures and functions of the brain by protecting it from external insults, and from pathogenic and toxic substances (Proulx 2021). Unfortunately, this protective effect makes them detrimental from the viewpoint of delivering drugs that target the CNS. During chemotherapy, for instance, the brain is known as a ‘sanctuary’ for extravasating malignant cells to escape from the anti-cancer drugs in the peripheral blood stream. These cells then lurk in the brain, eventually developing fatal metastases therein (Wilhelm et al. 2018). Efforts have been made to circumvent the BBB by administering enzymes to patients with neuronopathic LSDs via intrathecal (IT) or intracerebroventricular (ICV) routes, so that they can be delivered directly to the brain parenchyma via the CSF (Matsuoka et al. 2011; Muenzer et al. 2016). Also, drug delivery to the brain has been attempted by harnessing the innate transcytosis mechanism via transferrin (Friden et al. 1991; Couch et al. 2013; Sonoda et al. 2018; Kariolis et al. 2020) and insulin (Boado et al. 2014; Giugliani et al. 2018b) receptors on the brain capillary endothelium, without disrupting the physiological mechanism of the BBB, as by hyperthermia (Leuthardt et al. 2016) and ultrasound (Lipsman et al. 2018; Abrahao et al. 2019).

However, better understanding and therapeutic utilization of the BBB and the CSF are of paramount importance for the development of novel therapeutics to address CNS disorders in patients with neuronopathic LSDs. A wide range of issues need to be addressed, from clarifying the underlying pathogenesis of neurodegeneration, developing and manufacturing BBB-crossing enzymes, to evaluating the optimal drug effects against CNS symptoms in clinical trials. Recent research into the BBB and CSF has been delineating the complex physiological nature of these fundamental mechanisms, which are still far from being fully elucidated. This review attempts to summarize recent advances in our understanding of the BBB and the CSF which need to be taken into account during research aimed at developing effective treatments for neuronopathic LSDs.

## Blood-brain barrier

1. General structures and functions

**Fig. 1:**
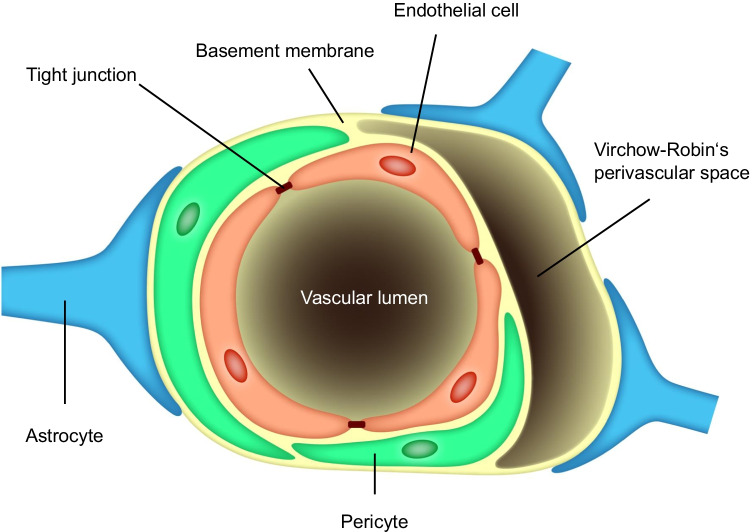
The neurovascular unit constituting the BBB (modified from Abbott et al. 2010; Brown et al. 2019)

Originally conceptualized by Lewandowsky (1900) following relevant observations by Ehrlich (circa 1885) and his contemporaries around the turn of the nineteenth century (Saunders et al. 2014), the term blood-brain barrier (or barrière hémato-encéphalique) was coined by Stern (1921) to describe a structure that prevents certain substances in the systemic circulation from entering the CNS and the CSF. Research in the 100 years since has clarified many of its functions and structures. As revealed by electron microscopic studies, it is located in the brain vasculature as a barrier between the blood and brain parenchyma, and it is maintained by intercellular tight junctions to limit paracellular and transcellular transport (Reese and Karnovsky 1967). Of late, the BBB is conceived of as a neurovascular unit, the major components of which comprise brain endothelial cells, pericytes, and astrocytes (Fig. 1). The neurovascular unit as a whole seems to regulate intracellular trafficking of substances in general, while it is the endothelial cells that play the major role in limiting entry of substances from the plasma into the brain (Fig. 1; Zhao et al. 2015; Villaseñor et al. 2019). The BBB is considered today to serve more broadly as an interfacing conduit that mediates communication and crosstalk between its two sides, namely the luminal (systemic circulation) and the abluminal (brain parenchyma) (Li et al. 2021). Through a series of physiological functions, the neurovascular unit actively controls vessel permeability and is vital for the maintenance of the CNS’s internal milieu and neural protection throughout a person’s life (Kadry et al. 2020; Profaci et al. 2020).

Dysfunction of the BBB is known to play a central role in a number of neurodegenerative diseases (e.g. Alzheimer’s disease and multiple sclerosis), whereby the disrupted BBB causes ion dysregulation and allows neurotoxic blood-derived debris, cells, and microbial pathogens to enter the brain; this leads to inflammation, opening up multiple possible pathways towards neurodegeneration (Sweeney et al. 2018, 2019).

(2) Transcytosis through the BBB – intracellular transport in cerebrovascular endothelial cells –

Table 1 summarizes various paracellular and transcellular pathways of potential value in delivering therapeutics and diagnostics across the BBB (Kopec et al. 2019).

**Table 1:** Pathways and methods for brain delivery of drugs and diagnostics (Kopec et al. 2019)

1) Transcellular pathways	-Passive diffusion through cell membranes in the BBB
	-Receptor-mediated transport through the BBB
	-Liposome-mediated drug delivery
	-Nanoparticle delivery
	-Exome-mediated delivery
	-Viral vector delivery
2) Paracellular pathways	-Osmotic delivery (hypertonic solution to disrupt the tight junctions)
	-Disruption of protein-protein interactions to increase the porocity of the tight junctions by claudin and occluding peptides

Perhaps due to the specialized tight junctions that prevent paracellular passage between the blood and the brain, transcellular pathways have been a major focus of attention as a means of drug delivery. In particular, transcytosis (i.e. the transport of molecules via vesicles through the microvascular endothelial cells in the CNS) is considered critical in overcoming the restrictive properties of the BBB (Villaseñor et al. 2019). There are two known types of transcytosis: 1) receptor-mediated transcytosis, whereby a ligand binding to receptors mediates endocytosis, as happens with insulin and transferrin, and 2) non-selective adsorptive transcytosis, in which charged interactions between the molecule and the plasma membrane of the endothelium facilitate its entry, as with albumin (Ayloo and Gu 2019). Of the two, innate receptor-mediated transcytosis is more selective and specific in ensuring exclusive entry of essential peptides and proteins into the brain on the one hand, and more effective in clearing toxic waste products from the brain into the blood on the other (Sweeney et al. 2018, 2019).

Transcytosis is initiated by endocytosis, a process by which substances in the luminal spaces are internalized by endothelial cells, and the internalization process can proceed via three pathways: the caveolae, clathrin-dependent endocytosis, or clathrin-independent endocytosis (Villaseñor et al. 2019). The intricate internalization process affects the efficiency of intracellular transport as a whole. In other words, because BBB permeability is dependent on the specific character and structures of particular transcellular pathways, these specifications are critical in harnessing transcytotic mechanisms for effective drug delivery. However, the transcellular pathways are more complex than initially supposed and have still not been fully clarified. Most research efforts have been devoted to paracellular pathways because of their associations with major neurodegenerative diseases (Preston et al. 2014). For instance, receptor-mediated transcytosis by way of transferrin and insulin receptors requires clathrin-dependent endocytosis, but clathrin-independent pathways may also be involved in receptor-mediated transcytosis. Thus, many details still remain to be clarified even with regard to receptor-mediated transcytosis, which is the transcytotic mechanism that has been most widely investigated for its potential application as a platform for drug delivery across the BBB (Sandvig et al. 2018), the other mechanisms being adsorptive-mediated transcytosis and carrier-mediated transcytosis (Terstappen et al. 2021).

(3) Drug delivery to the brain via receptor-mediated transcytosis

The possibility of utilizing specific transcytosis for drug delivery to the CNS has had a notable translational impact on drug development (Ayloo and Gu 2019). Indeed, after more than 25 years of failed attempts to develop biologics for brain diseases, the possibility of using transcytotic mechanisms to reengineer biologics has been greeted with great excitement, and has even been touted as a molecular Trojan horse (Pardridge 2017, Bellettato and Scarpa 2018) in reference to the use of a monoclonal antibody (MAb) against an endogenous BBB receptor transporter, which is expected to serve as a molecular shuttle to enhance the delivery of biologics to the brain (Goulatis and Shusta 2017).

To date, four groups of receptors have been targeted for potential use in receptor-mediated transcytosis (Terstappen et al. 2021):

1. Transferrin receptors
2. Insulin and IGF receptors
3. LDL-receptor family: LDL receptors, LDLR-related protein 1, LDLR-related protein 8, transmembrane protein 30A, melanotransferrin
4. Solute carrier transporters: CD98 heavy chain, SLC2A1

Among these, transferrin and insulin receptors have been in the limelight because of their utilization in BBB-crossing enzyme replacement therapy (ERT) for neuronopathic LSDs. Five compounds that harness receptor-medicated transcytosis have so far progressed to clinical trials (Table 2).

**Table 2:** Clinical trials of drugs for neuronopathic MPS that utilize receptor-mediated transcytosis

Disease	Compound	Clinical phase/status	Targeted receptor	Sponsor	Publication	Identifier
MPS I	AGT-181 (valanafusp alpha)	Phase I (completed)	Insulin receptor	ArmaGen	Giugliani et al. (2018b)	NCT02262338
	JR-171	Phase I (recruiting)	Transferrin receptor	JCR Pharmaceuticals	Not available	NCT04227600
MPS II	AGT-182	Phase I /II (completed)	Insulin receptor	ArmaGen	Not available	NCT03053089
	JR-141 (Pabinafusp alfa)	Phase III (completed) Approved in Japan	Transferrin receptor	JCR Pharmaceuticals	Okuyama et al. (2019, 2021) Giugliani et al. (2021a, b)	NCT03568175
		Phase III (Recruiting in US, EU and Brazil)			Not available	NCT04573023
	DNL-310	Phase I/II (recruiting)	Transferrin receptor	Denali Therapeutics	Not available	NCT04251026

**Fig. 2:**
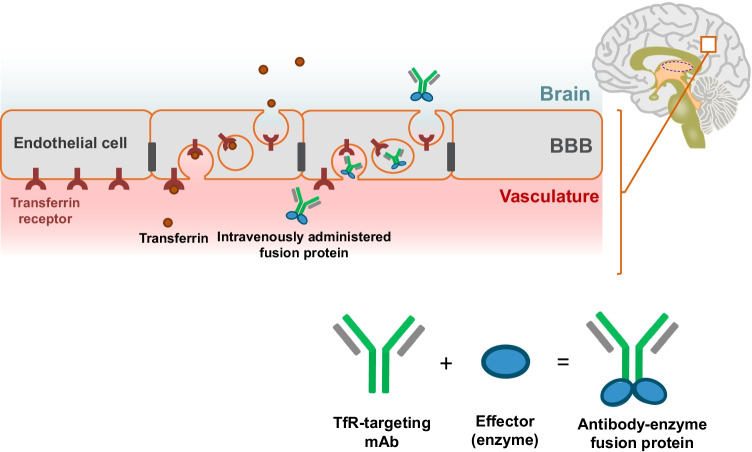
Receptor-mediated transcytosis for drug delivery across the BBB

As an example, a schematic illustration showing the mechanism of transcytosis via transferrin receptors is given in Fig. 2.

Transferrin binds to the transferrin receptors located on the luminal side of the microvascular endothelial cells in the brain and is absorbed into the endothelial cells (endocytosis), in which it is then transported towards the abluminal side of the cell facing the brain parenchyma, and subsequently released from the receptors to reach the brain parenchyma (exocytosis). Likewise, enzymes fused with an anti-transferrin receptor antibody bind to the transferrin receptors, are absorbed into the endothelial cells, and are then released into the abluminal side of the cells so that they can diffuse into the brain parenchyma.

There have been a number of translational challenges in establishing drug delivery via receptor-mediated transcytosis. One of them is that using receptor-mediated transcytosis of targeted enzymes can affect the original innate transcytosis that is supposed to deliver physiological substances like transferrin (Bien-Ly et al. 2014; Sonoda et al. 2018; Ullman et al. 2020) and insulin (Boado et al. 2014).

A clinical trial carried out to evaluate the safety and efficacy of AGT-181 (valanafusp alpha), α-L-iduronidase fused with anti-insulin receptor antibody, in patients with MPS-I detected drug-related transient hypoglycaemia in 6.4% of the patients (Giugliani et al. 2018b). However, this finding can be attributed to the weak insulin agonist activity of the anti-insulin receptor antibody that constitutes AGT-181 (Boado et al. 2012), and is not necessarily related to the transcytotic effect per se of the compound.

During three clinical trials of pabinafusp alfa (JR-141), iduronate-2-sulfatase fused with anti-transferrin receptor antibody, on patients with MPS-II, there were no adverse events associated with the iron metabolism, which it was thought the drug might affect (Okuyama et al. 2019, 2021; Giugliani et al. 2021a, b). This finding was buttressed by a nonclinical safety evaluation which found that pabinafusp alfa had no inhibitory effect on the interaction of transferrin with its receptors (Yamamoto et al. 2021).

In a clinical trial of DNL-310, an enzyme fusion protein that contains a low-affinity transferrin-binding peptide, anaemia was detected in two of the five patients with MPS II administered with DNL-310, although this was considered to be unrelated thereto (Watts and Ho 2021). However, potential association of DNL-310 with anaemia in relation to the transferrin receptors, also expressed in high amounts on erythroblasts, may require further evaluation.

Overall, these clinical data suggest that the antibodies targeting the innate receptors associated with transcytosis may potentially lead to receptor-associated adverse events, but the incidence and clinical significance of the events observed so far vary from minimal to negligible. The potential risks of these events are almost certainly outweighed by the clinical benefits of harnessing receptor-mediated transcytosis to deliver drugs to the CNS to treat the severe neurodegeneration that has hitherto been impossible to address.

Another translational challenge is how to optimize the affinity of the anti-receptor antibody to achieve efficient brain delivery, biodistribution, and pharmacodynamics of a lysosomal enzyme fused with an antibody (Arguello et al. 2021). Monovalent transferrin receptor (TfR) antibodies with lower receptor affinity are reported to achieve greater brain penetration than bivalent TfR antibodies with high affinity (Bien-Ly et al. 2014; Arguello et al. 2021). However, a recently published mathematical model of TfR-mediated transcytosis across the BBB (Pardridge and Chou 2021) concludes that antibodies with a relatively high affinity can achieve efficient transport across a wide range of injection doses, whereas antibodies with a low affinity enable transport only at a high dose, suggesting a series of complex correlations involving antibody affinities, required dosages, and brain penetration.

The selection of epitopes on the receptor for optimal transcytosis is also an important issue in need of further clarification. It has a significant impact on endocytosis and the subsequent trafficking of a molecule after it binds to, and is internalized in, the target receptor (Kim et al. 2020), meaning epitopes are critical for transcytosis as a whole.

Yet another complex matter is the affinity optimization of the BBB-receptor targeting moieties, because such hybrid fusion proteins contain one or more sites for cellular binding and uptake other than the TfR-binding moiety. For example, the mannose-6-phosphate (M6P)-containing glycans on the lysosomal enzyme that mediate enzyme uptake by the M6P receptor are present on most cells in the body; hence the resultant somatic efficacy of the fusion protein. The affinity of the M6P-containing glycans to the M6P receptor is in the low nM range, ensuring rapid cellular uptake of the enzyme (Gary-Bobo et al. 2007; Kanzaki et al. 2020). To optimize enzyme uptake into the CNS and non-CNS organs alike, the affinity ranges to the M6P receptor and the BBB-targeting receptor should be of a similar magnitude, or the latter should have a slightly higher magnitude than the former, as a disproportionately low affinity of fusion protein to the BBB-targeting receptor can compromise brain delivery of the enzyme. Compensation of low affinity to the BBB-targeting receptor by higher dosing, though plausible in theory, would actually be counterproductive, as it could cause immunogenicity issues during long-term treatment.

In summary, safe and efficient transcytosis requires optimization of multiple factors, including the valency and receptor affinity of the antibodies involved, the affinity ratio between the BBB-targeting moiety and M6P receptor-targeting glycans, the binding epitopes on the BBB-targeting receptors, the molecular structure of the fusion protein as a whole, molecular retention in the systemic circulation, and actual dosage regimen. All of these issues have to be resolved before transcytosis can be safely used clinically.

## The cerebrospinal fluid (CSF) and its circulation

1. Evolving perspectives on CSF circulation

The CSF provides buoyancy and cushioning to the brain and the spinal cord to protect them from physical injury, and it preserves solute concentrations and intracranial pressure (Naseri Kouzehgarani et al. 2021). The CSF is also a rich source of nutrients, hormones, neurotrophic factors, growth factors, and other signalling molecules, which it circulates throughout the brain parenchyma to maintain CNS homeostasis (Fowler et al. 2020; Proulx 2021). Furthermore, the CSF functions as a sink for brain extracellular solutes (Iliff et al. 2012), which, through the CSF, will then be drained into the systemic blood and lymphatic circulation.

**Fig. 3:**
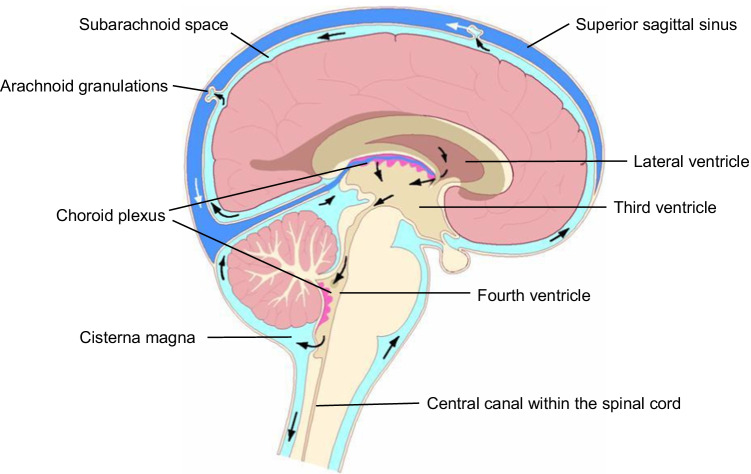
The cerebospinal fluid circulation (Benveniste et al. 2017; Naseri Kouzehgarani et al. 2021)

The traditional view is that most CSF is secreted by the choroid plexuses in the cerebral ventricles, is circulated, and is then finally drained into the venous blood or the lymphatic system. Three components thus play a key role in CSF physiology: 1) active CSF formation (secretion), 2) passive CSF absorption (drainage), and 3) unidirectional CSF flow from its formation sites to absorption sites (Fig. 3).

A new hypothesis regarding the CSF microcirculation (also known as the glymphatic [glial-lymphatic] system) that is almost antithetical to the traditional view has recently been put forth (the Bulat-Orešković-Klarica hypothesis; Oresković and Klarica 2010; Bulat and Klarica 2011; Liff et al. 2015; Natale et al. 2021). According to this hypothesis, CSF secretion and absorption are not localized as in the traditional view, but occur everywhere in the CSF circulatory pathway. The primary source of CSF is considered to be the fluid flow between the capillaries and the interstitium, while the choroid plexus, accordingly, contributes less to CSF generation than the traditional view holds. The new hypothesis postulates that CSF circulation is bidirectional throughout the entire CSF system, and that large molecules are able to reach the brain parenchyma via the glymphatic pathway, provided that CSF clearance is sufficiently counteracted to achieve steady-state concentrations of the substances in the subarachnoid space for a sustained period (Naseri Kouzehgarani et al. 2021). In other words, CSF microcirculation is considered to involve the brain parenchyma, into which CSF circulates through the perivascular spaces (or Virchow-Robin spaces) of the penetrating arteries and then enters through the astrocyte endfeet surrounding the microvasculature via aquaporin-4 water channels. The CSF then mixes with the interstitial fluid and enters the perivascular space of the exiting venulae via the astrocyte endfeet, and flows back into the subarachnoid space (Rasmussen et al. 2018; Nakada and Kwee 2019). Though this hypothetical model may shed new light on the pharmacokinetics and pharmacodynamics of large molecules in the CSF, as the exact mechanism of the glymphatic microcirculation (Mestre et al. 2020) as well as the details of CSF outflows in general (Proulx 2021) remain controversial, uncertainties still surround the pharmacokinetic and pharmacodynamic implications of the glymphatic pathway.

2. CSF circulation and drug distribution: the pharmacokinetic perspective

**Fig. 4:**
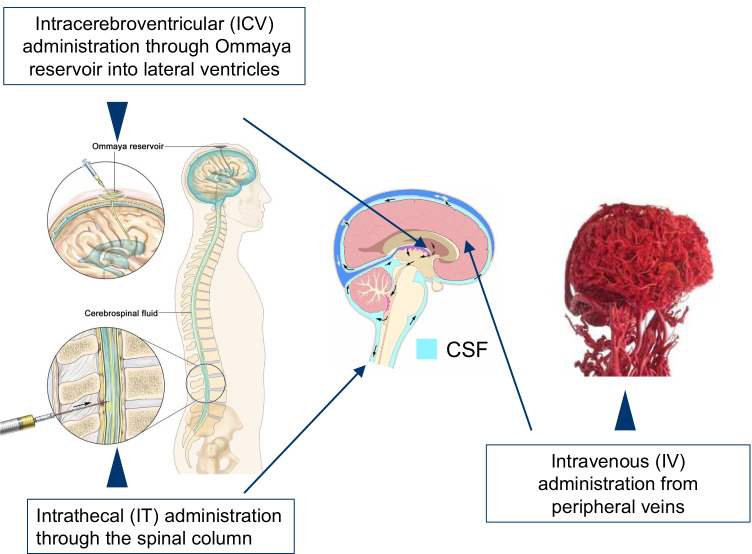
Routes of drug administration into the CNS

Attempts have been made to administer enzymes deficient in neuronopathic LSDs directly into the CSF, so that they can be distributed via the CSF flow and reach the brain parenchyma (intra-CSF administration; Naseri Kouzehgarani et al. 2021). In this approach, the CSF assumes a pharmacokinetic role by providing a critical route for drug distribution in the CNS. Intra-CSF administration can be made by IT administration, whereby the enzyme is directly injected via lumbar puncture into the subarachnoid space. ICV and intra-cisterna magna (ICM) injections are also reported to achieve similar drug delivery into the CSF (Fowler et al. 2020). Figure 4 illustrates these intra-CSF administrations along with intravenous administrations.

Because CSF flow in and out of the brain is highly regulated (Kouhi et al. 2021), appropriate distribution of enzymes administered via IT, ICV, or ICM routes needs to be ensured to enable and sustain effective therapeutic concentrations. Indeed, drugs injected into the CSF compartment are known to be rapidly transported out of the brain into the blood, while drug penetration into the brain parenchyma from the CSF is considered minimal (Pardridge 2011). According to the traditional view of a unidirectional CSF circulation, this is due to the expected retrograde (i.e. cephalad or headward) diffusion of the drug through the subarachnoid space and the ventricles to reach the brain parenchyma. The inadequacy of drug distribution via the CSF may potentially be compensated for by nanomedicine systems, which can navigate the subarachnoid space to sustain delivery of therapeutic molecules, genes, and imaging agents within the CNS (Fowler et al. 2020).

Of late, ICV administration has been suggested as a viable drug delivery route to the brain parenchyma, as exemplified by the ICV administration of cerliponase alfa for the treatment of neuronal ceroid lipofuscinosis type 2 (Schulz et al. 2018; de Los Reyes et al. 2020). However, a number of constraints mean that even after successful drug distribution following intra-CSF administration to the inner brain parenchyma, significant drug efficacy is not fully guaranteed (Naseri Kouzehgarani et al. 2021). For instance, as the glymphatic pathway is known to be primarily active during sleep, it is not clear to what extent it can be utilized as a stable route for drug administration to patients when they are awake (Mestre et al. 2020). Hence, using the CSF as a vehicle for drug delivery is still considered problematic (Abbott et al. 2018). Compared with intravenous administration of BBB-crossing enzymes, it seems highly unlikely that intra-CSF administration aimed at distributing a drug across the pia mater will achieve diffuse intraparenchymal penetration. The deep vascularization of the brain should allow efficient delivery of intravenously administered drugs to the microvasculature, followed by receptor-mediated transcytosis and resultant extensive diffusion.

A practical point with important clinical relevance is that intra-CSF administration cannot, by definition, address the multiple somatic symptoms caused by the peripheral substrate accumulations seen across all LSDs, which suggests that patients with both CNS and peripheral/somatic symptoms would need to undergo intra-CSF administration and conventional intravenous ERT simultaneously on a long-term basis. This would inevitably add to the clinical onus on pediatric patients with neuronopathic LSDs and lead to frequent complications (Cohen-Pfeffer et al. 2017; Slavc et al. 2018) caused by long-term intra-CSF administrations via a catheter. Intravenous administration of BBB-crossing enzymes, thanks to their dual efficacy against both CNS and peripheral symptoms, obviates the burden of concomitant administrations, and can be considered conducive to long-term treatment.

These issues may explain the still limited successful use of intra-CSF drug delivery, despite the large number of clinical trials employing this method to address CNS diseases (Naseri Kouzehgarani et al. 2021). Thus, although intra-CSF drug administration has been shown to be a viable delivery route, it still involves a number of contingent issues that need to be overcome before it can be widely applied for safe and effective treatment.

3. The CSF and its pharmacodynamic role in efficacy evaluation

Intracerebral concentrations of the substrates (e.g. heparin sulfate [HS]) that remain uncatabolized because of the enzyme deficiency seen in patients with neuronopathic LSDs are by far the most important determinant in initiating the complex cascade of neurodegenerative processes (Sato and Okuyama 2020). As the monitoring of the intracerebral substrate levels is highly invasive and unjustifiable, substrate levels in the CSF have instead been measured in MPS I (Raymond et al. 2016) and MPS II (Hendriksz et al. 2015), while measurement of undigested substrates in the urine have been an established laboratory diagnostic procedure for the detection of MPS patients in clinical practice.

In an established animal model of MPS II, close correlations were observed between intracerebral and CSF HS levels, and intravenous administration of pabinafusp alfa led to dose-dependent reductions in HS levels in the CSF, along with corresponding reductions in HS levels in the brain (Sonoda et al. 2018; Tanaka et al. 2018). These reductions in substrate levels led to significantly alleviated neurodegeneration, as evidenced by histopathological improvements and preserved neurocognitive functions (Morimoto et al. 2021). CSF substrate levels are, therefore, not only an important diagnostic biomarker of disease activity, but can also constitute, when validated appropriately, a reliable and sensitive efficacy endpoint by which to evaluate the efficacy of therapeutics that can cross the BBB against CNS symptoms. Indeed, there is mounting clinical evidence of a correlation between HS levels in the CSF and disease severity in patients with neuropathic MPS (Okuyama et al. 2021; Giugliani et al. 2021a, 2021b; Tomita et al. 2021). Similarly, a correlation between reduced HS levels in the CSF and improvements in CNS manifestations has also been demonstrated (ibid.). In this way, the CSF has been revealed to play a useful pharmacodynamic role in providing a parameter by which the efficacy of potential therapeutics against the CNS manifestations of neuronopathic LSDs can be evaluated. Moreover, changes in substrate concentrations in the CSF can be used to monitor individual patients’ responses to ERT against CNS manifestations, so that treatment can be optimized individually for patients with neuronopathic LSDs with enormous phenotypic heterogeneity.

**Fig. 5:**
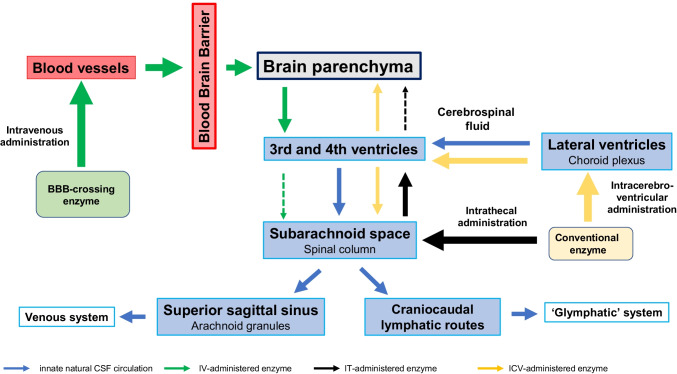
Cerebrospinal fluid circulation and drug delivery

IT administration of enzymes in patients with MPS-II (Muenzer et al. 2016; NCT02055118) and MPS IIIA (Jones et al. 2016) leads to significant reductions in CSF HS levels, but does not show corresponding CNS efficacy. In contrast, ICV administration of idursulfase beta both decreases CSF HS levels and produces positive neurodevelopmental changes (Seo et al. 2021). These mixed results may, prima facie, call into question the clinical validity of CSF HS levels as an efficacy endpoint. However, caution is needed in using CSF substrate levels as a surrogate of brain substrate levels when drugs are injected directly into the CSF, because reductions in substrate levels in the compartment to which the enzyme is administered cannot be assumed to indicate reductions in substrate levels in the brain. Certainly, further clarification is needed of the implications and interpretations of substrate concentrations in the CSF in association with the different administration routes. Figure 5 show the relationships between CSF circulation and different administration routes of enzymes used to treat neuropathic LSDs.

The blue arrows represent the innate natural circulation of the CSF, the green arrows intravenously administered BBB-crossing enzymes, the black arrows intrathecally administered conventional enzymes, and the yellow ones intracerebroventricularly administered conventional enzymes (Modified from Tumani et al. 2017; Shapey et al. 2019).

IT administration of enzymes can, by definition, directly affect the substrate levels in the subarachnoid space, while their penetration into the brain parenchyma to act against CNS disorders is very limited, as discussed above. In contrast, BBB-crossing enzymes administered intravenously can traverse the BBB via receptor-mediated transcytosis, thus addressing the brain parenchyma widely and directly, where they reduce the substrate accumulations therein. The enzymes are thought to be mostly consumed in the process, so that few, if any, remnants circulate into the CSF. Therefore, changes in CSF substrate levels brought about by intravenous ERT with BBB-crossing therapeutics truly reflect the changes in intracerebral substrate levels.

As shown in Fig. 5, ICV administration is believed to deliver larger amounts of enzymes to the brain parenchyma than IT administration, so drug efficacy against CNS symptoms is demonstrated more distinctly with ICV administration. In other words, although IT-administered enzymes can exert direct effects on the substrates in the subarachnoid space and bring about significant reductions, they do not appear to reach and diffuse throughout the brain parenchyma in sufficient quantities to significantly affect the intracerebral substrate accumulations, because, according to the traditional view, drug distribution following IT administration would have to go against the unidirectional CSF flow. Consequently, the implications of CSF substrate levels following IT administration of enzymes should not be equated with those following ICV administration, because the physiologies *sui generis* of the CSF and BBB have to be taken into account when evaluating the pharmacokinetics and pharmacodynamics of drugs targeting the CNS.

## Conclusion

The year 2021 marks the centenary of the naming of the BBB by Lina Stern (1878–1951; Dreifuss and Tikhonov 2007), and subsequent research has revealed its far-reaching importance. It may seem to be counterintuitive serendipity that something that has long been regarded as an impermeable barrier between the brain and the blood is now being harnessed as a hitherto unrecognized gate to deliver drugs to treat CNS disorders that were previously impervious to pharmacotherapy, without compromising the BBB’s original function as a barrier. Furthermore, rapidly expanding research on the CSF has not only given us a better understanding of this well-known innate mechanism but has also identified a new role for it in treating neuronopathic LSDs. Likewise, research efforts to develop novel therapeutics for these diseases have in turn contributed to our understanding of these two essential mechanisms to protect the CNS, and have revealed the pathological implications of the BBB for a number of neuropathologies (Sweeney et al. 2018).

However, there are still a number of remaining issues surrounding the emerging ERTs for neuronopathic LSDs. First, details of the specific regulating processes in various modes of transcytoses need to be clarified so that drug delivery via transcytosis can be optimized to consolidate efficiency, efficacy, and safety. Second, the CSF microcirculation has only been partly elucidated, and its pharmacokinetic and pharmacodynamic relevance to ERT and other treatment modalities for neuronopathic LSDs needs further investigation. Third, knowledge about how the neuronopathy seen in patients with LSDs responds to treatment is still scant, which means that valid and reliable methods of evaluating the efficacy of novel therapeutics are yet to be established. In other words, with the advent of new therapies to address the neurological and psychiatric signs and symptoms of patients with neuronopathic LSDs, we need to learn to understand how these new treatments influence an affected individual in the way he/she feels, functions, and survives. Also, the inevitable paucity of patient populations and significant heterogeneity in the clinical manifestations and prognoses of these rare diseases impede the efficient conduct of clinical trials and cause further delays in introducing novel treatments. Last but not least, designing and manufacturing a fusion protein for efficient transcytosis-mediated drug delivery entails formidable challenges in establishing acceptable pharmacokinetic and pharmacodynamic profiles, stability, yield, and other production-related technical specifications.

It is hoped that the wide-ranging research being carried out will lead to further insights into neuronopathic LSDs, facilitating the development of effective treatments for patients suffering from these rare, debilitating diseases.

## Data Availability

not applicable.
